# Clinical Indicators of Bone Deterioration in Alcoholic Liver Cirrhosis and Chronic Alcohol Abuse: Looking beyond Bone Fracture Occurrence

**DOI:** 10.3390/diagnostics14050510

**Published:** 2024-02-28

**Authors:** Milos Stulic, Jelena Jadzic, Natasa Dostanic, Milica Zivkovic, Tihomir Stojkovic, Jelena Aleksic, Stefan Stojkovic, Milica Stojkovic Lalosevic, Marko Vojnovic, Zeljko Vlaisavljevic, Jelena Martinov Nestorov, Tatjana Nikolić, Violeta Culafic Vojinovic, Djordje Culafic, Danijela Djonic

**Affiliations:** 1 Clinic for Gastroenterohepatology, University Clinical Center of Serbia, 11000 Belgrade, Serbia; drstulic@gmail.com (M.S.); stefanstojkovic@ymail.com (S.S.); jelenamartinov@yahoo.com (J.M.N.);; 2 Faculty of Medicine, University of Belgrade, 11000 Belgrade, Serbia; 3 Center of Bone Biology, Faculty of Medicine, University of Belgrade, 11000 Belgrade, Serbia; jelena.jadzic@med.bg.ac.rs; 4 Special Hospital for Addiction Diseases “Drajzerova”, 11000 Belgrade, Serbia; nakidostanic@gmail.com; 5 Institute of Medical and Clinical Biochemistry, Faculty of Medicine, University of Belgrade, 11000 Belgrade, Serbiatatjana.nikolic@med.bg.ac.rs (T.N.); 6 Institute for Health Protection of Workers of Serbian Railways, 11000 Belgrade, Serbia; jelenavasic1974@gmail.com; 7 Euromedik Hospital, 11000 Belgrade, Serbia

**Keywords:** alcoholic liver cirrhosis, chronic alcohol abuse, osteoporosis, FRAX, trabecular bone score, dual-energy x-ray absorptiometry (DXA), hip structure analysis, bone turnover biomarkers, men

## Abstract

Although previous studies indicated that chronic alcohol abuse (CAA) and alcoholic liver cirrhosis (ALC) are associated with increased bone fragility, understanding bone fragility determinants is still modest in these individuals. We used a comprehensive individualized clinical fracture risk assessment approach (vertebral osteodensitometry, femoral osteodensitometry and geometry, and serum bone turnover biomarkers) to compare adult male patients with ALC who have not previously had femoral or vertebral fractures (*n* = 39), patients with CAA (without liver cirrhosis, *n* = 78) who have not previously had femoral or vertebral fractures and healthy age- and sex-matched controls (*n* = 43). Our data suggested that intertrochanteric bone mineral density was significantly lower in ALC and CAA patients than in controls. Also, the trabecular bone score was considerably lower in ALC patients compared with CAA and control individuals. The most significant inter-group differences in femoral geometry were noted on the femoral shaft. Patients with ALC and CAA have a higher 10-year risk of major osteoporotic fractures compared to the controls. Analysis of bone turnover biomarkers showed increased osteoprotegerin and beta-C-terminal telopeptide serum concentrations and decreased insulin growth factor-1 concentrations in patients with ALC compared to CAA and control groups. Our data revealed that bone alterations are present in patients with ALC and CAA even if they did not sustain a nontraumatic bone fracture, but it is also indicative that current bone-assessing clinical methods are not entirely reliable. Thus, future studies should focus on developing a reliable integrative clinical tool that can be used to accurately predict and prevent bone fracture occurrences in patients with ALC and CAA.

## 1. Introduction

Chronic alcohol abuse (CAA) has been known to significantly increase the risk of developing more than 200 different diseases, including chronic liver disease (CLD) [1,2]. In the last two decades, the liver-disease-related mortality rate has been steadily growing worldwide. It is estimated that around two million people die annually due to complications associated with the irreversible stage of CLD [3]. Apart from the increased mortality rate, the high economic impact and significantly poor quality of life in patients with CAA and CLD represent a significant problem in the clinical management of these individuals [4,5]. Although CLD and CAA are associated with numerous complications that affect other organs, the most neglected complications are skeletal alterations [6] and the increased risk of sustaining nontraumatic vertebral and hip fractures [7,8]. It is important to point out that the frequency of osteopenia and osteoporosis is up to one-half of patients with alcoholic liver cirrhosis (ALC) and CAA [9], and the shift toward fracture occurrence in younger ages has been noted in these individuals [8]. Since osteoporosis and bone fragility are more frequently observed in postmenopausal women, and these disorders are more frequent in men [10], it is important to note that ALC and CAA could change the sex distribution of fracture risk in the population. Also, a particularly worrying observation is that the mortality rate associated with hip fracture in patients with ALC was 2.8-fold higher compared to the general population [8], indicating the urgent need for adequate bone-assessing tools in the clinical management of these patients [11].

Several pathogenetic mechanisms could contribute to bone fragility in individuals with CAA and alcohol-induced CLD, with the most prominent effect in patients with ALC. Except for the general mechanisms (hypogonadism, poor nutritional status, and low levels of vitamin D), the direct toxic effect of alcohol and the strong link between systemic hyperproduction of inflammatory mediators and increased bone resorption could be among the reasons for bone loss in these individuals [6,12,13]. Nevertheless, numerous studies confirm that the process that leads to CLD-induced bone impairment is multifactorial and still insufficiently understood, warranting further research.

So far, numerous studies have been conducted to analyze bone fragility determinants in patients with ALC and those with CAA who do not have liver cirrhosis. However, results obtained from clinical fracture risk assessments are inconsistent and often conflicting [9,14,15,16,17,18]. Comparability of these data is limited since previous studies substantially differ in their methodological settings and study design (criteria for inclusion in control group, different stages of liver disease, mixed male and female sexes, limited number of individuals, patients with and without fractures). Besides, according to our knowledge, a study combining a comprehensive and individualized approach in clinical fracture risk assessment is absent in ALC patients and individuals with CAA, especially those without previous bone fractures. Furthermore, bone changes in those patients remain a topic for further investigation to generate unique guidelines for adequate diagnosis and treatment modalities [19].

Bearing in mind all previously stated, this cross-sectional study aimed to conduct a comprehensive and individualized clinical fracture risk assessment in male patients with ALC and CAA (without liver cirrhosis) who did not previously sustain femoral and vertebral nontraumatic fractures. Thus, we aimed to compare osteodensitometry parameters of lumbar vertebrae, osteodensitometry and bone geometry parameters of various subregions of proximal femora, and serum concentrations of specific bone turnover biomarkers in each individual included in our ALC, CAA, and control group.

## 2. Materials and Methods

### 2.1. Patient Recruitment

**Figure 1:**
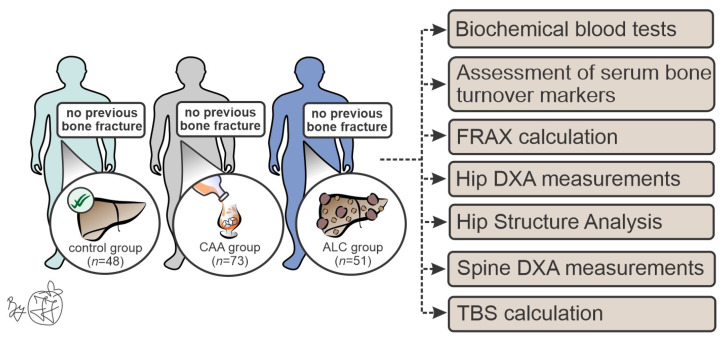
Methodological approach in our study. Abbreviations: CAA—chronic alcohol abuse; ALC—alcoholic liver cirrhosis; FRAX—Fracture risk assessment tool; DXA—dual-energy X-ray absorptiometry; TBS—trabecular bone score.

This cross-sectional study included a total of 172 male patients who were divided into three groups (Figure 1): male patients with ALC (ALC group, *n* = 48), male patients with chronic alcohol abuse without liver cirrhosis (CAA group, *n* = 73), and healthy age-matched male individuals (control group, *n* = 51). We applied the following exclusion criteria: female individuals, positive history of endocrine and metabolic skeleton-affecting diseases (such as parathyroid and thyroid dysfunction, hypogonadism, type 2 diabetes mellitus, autoimmune, hereditary, or viral CLD), the presence of solitary and/or metastatic malignant lesions, the usage of bone turnover-affecting drugs (antiepileptics, cytostatics, corticosteroids, vitamin D, bisphosphonates), intravenous drug-abuse, as well as state of permanent immobility. To exclude individuals with femoral and vertebral nontraumatic fractures, initially, all patients were subjected to radiography of both hips and the vertebral column. During our recruitment period, six patients with ALC who initially fulfilled the inclusion criteria had a nontraumatic vertebral fracture, indicating that those individuals should be excluded from further analyses. No vertebral fractures were verified in the CAA and control group, nor were hip fractures noted in all included subjects. After biochemical analyses and ultrasound examination, subjects with findings that suggest metabolic/endocrine dysfunctions or any pathological finding in the abdomen were excluded from the control group.

The ALC group consisted of patients with ALC, hospitalized in the Clinic for Gastroenterohepatology, University Clinical Center of Serbia, who have been in stable abstinence from alcohol abuse for at least one year. These patients’ average age, height, weight, and body mass index (BMI) were as follows: 51.96 ± 7.56 years, 176.36 ± 8.09 cm, 85.95 ± 14.03 kg, and 27.64 ± 4.18 kg/m^2^. The diagnosis of ALC was based on a detailed history of alcohol consumption and previous medical documentation (50 g of pure alcohol/day for more than five years), clinical signs (physical stigmata of CLD, indirect signs of portal hypertension verified by abdominal ultrasound and/or endoscopy) and serum biochemical analyzes of hepatocyte integrity, enzymes of cholestasis and synthetic liver function.

The CAA group consisted of individuals with CAA without liver cirrhosis who were hospitalized in the Special Hospital for Addiction Diseases and fulfilled the criteria of the National Institute on Alcohol Abuse and Alcoholism (NIAAA). These individuals consumed alcohol until the day of admission to treatment (drinking four alcoholic drinks a day, or 14 drinks per week). The average age, height, weight, and BMI in the CAA group were 49.37 ± 8.82 years, 176.97 ± 7.31 cm, 80.65 ± 12.34 kg, and 25.68 ± 3.20 kg/m^2^, respectively.

The control group included age-matched male patients in whom hemorrhoidal disease was confirmed endoscopically without any other pathological findings. The average age, height, weight, and BMI of control individuals were as follows: 49.93 ± 11.92 years, 180.75 ± 5.95 cm, 92.25 ± 16.16 kg, and 28.24 ± 4.84 kg/m^2^.

### 2.2. Biochemical Blood Tests and Serum Biomarkers of Bone Metabolism

All study participants had standard biochemical blood tests (blood cell count, hemostasis, biochemistry), analysis of sex hormones (total and free testosterone, estradiol, luteinizing hormone—LH, follicle-stimulating hormone—FSH, dehydroepiandrosterone sulfate—DHEAS and sex hormone-binding globulin—SHBG), and analysis of vitamin D, parathyroid hormone (PTH) and osteocalcin concentrations. Blood was consistently sampled in the morning after all-night fasting. After blood sampling in a vacutainer without additives, centrifugation at 3500 rpm, and separation, the sample was stored in a deep freezer (−80 °C). We also analyzed specific bone turnover biomarkers (beta-C-terminal telopeptide—β-CTX, receptor activator of nuclear factor kappa beta ligand—RANKL, insulin-like growth factor 1—IGF-1 and osteoprotegerin—OPG) using adequate commercially available ELISA kits (Abcam plc, Cambridge, UK and Abbexa, Cambridge, UK). In accordance with the manufacturer’s instruction manuals, storage and analysis of blood samples were conducted at the Institute of Medical and Clinical Biochemistry, Faculty of Medicine in Belgrade.

### 2.3. Osteodensitometric Measurements and Hip Structural Analysis (HSA)

Osteodensitometric measurement of the proximal femora and lumbar spine was performed using dual-energy X-ray absorptiometry (DXA) on a HOLOGIC 1000 W device (Hologic QDR 1000/W; Hologic, Waltham, MA, USA). Using standard Hologic APEX software (version 2.0, Bedford, MA, USA), bone mineral density (BMD; g/cm^2^) was determined in the standard subregions of the proximal femora and the lumbar spine. Based on these data, the fracture risk assessment tool (FRAX) and trabecular bone scores (TBS) were calculated for all included individuals. The geometric femoral parameters were determined using a special built–in HSA software (version 2.0, Bedford, MA, USA) in standard regions of interest (narrow neck, intertrochanteric region, and femoral shaft). The following parameters were calculated for each region: periosteal diameter (PD; cm), endocortical diameter (ED; cm), cross-sectional area (CSA; cm^2^), cross-sectional moment of inertia (CSMI; cm^4^), sectional modulus (SM; cm^3^), cortical thickness (Ct.Th; cm), and buckling ratio (BR; dimensionless).

### 2.4. Ethical Considerations

The Ethics Committee of the Faculty of Medicine, University of Belgrade, confirmed that the study was conducted by the Guidelines for Good Clinical Practice, the Declaration of Helsinki, and local laws and regulations (approval no. 1322/IX-11). The study protocol was approved by the Joint Research and Ethics Committee (University Clinical Center of Serbia, approval no. 890/9; Special Hospital on Addiction, approval no. 2964). Written informed consent was obtained from all participants included in the study.

### 2.5. Statistical Analysis

The Kolmogorov−Smirnov test was used to assess the data distribution normality. Levine’s test was used to determine the data homogeneity before conducting an analysis of covariance (ANCOVA) with Bonferroni posthoc correction to estimate intergroup differences in mean values of the examined osteodensitometry parameters (covariates appearing in the corrected model were evaluated at a BMI value of 27.73 kg/m^2^). Analysis of variance (ANOVA) with Bonferroni posthoc correction was conducted to assess the significance of the difference in biochemical blood parameters and bone turnover biomarkers between the ALC, CAA, and control groups. All parameters that did not follow normal distribution were analyzed by adequate nonparametric tests (Kruskal−Wallis and Mann−Whitney tests). Statistical analyses were conducted using SPSS statistical software (version 21, IBM Corp, Armonk, NY, USA) at a significance level of 5% (0.05).

## 3. Results

### 3.1. Biochemical Analyses

As expected, biochemical analyses of blood samples from patients with ALC revealed affected synthetic and excretory liver function. Furthermore, biochemical analysis of parameters of bone metabolism showed significantly lower concentrations of total and ionized calcium in the ALC group, while the concentrations of phosphorus, vitamin D, and PTH were substantially lower in patients with ALC, as well as in the CAA group, compared to the control group. The analysis of sex hormones showed significantly lower values of LH and SHBG in both examined groups compared to the control, while free testosterone was reduced only in the ALC group (Table 1).

### 3.2. Osteodensitometry and Geometric Parameters of Proximal Femora and Lumbar Spine

**Figure 2:**
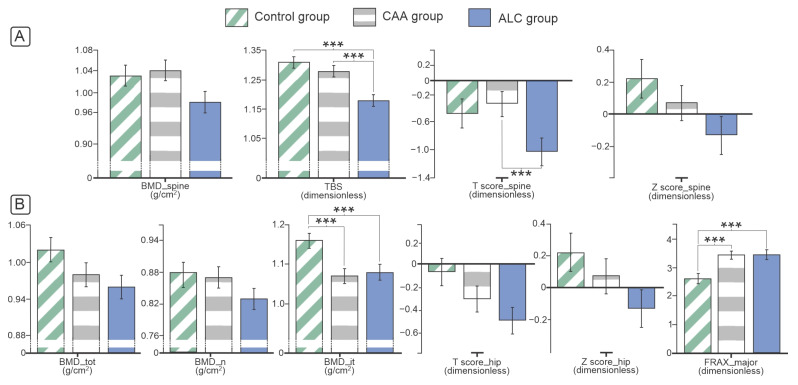
Vertebral (**A**) and femoral osteodensitometry (**B**) in ALC and CAA individuals. Analysis of covariance (ANCOVA) with Bonferroni posthoc correction was used for statistical analysis of the difference in BMI-adjusted parametric data (bar charts present data as mean ± standard error, *** marking significant difference). Abbreviations: ALC—alcoholic liver cirrhosis, CAA—chronic alcohol abuse, BMD—bone mineral density, TBS—Trabecular bone score, FRAX—Fracture Risk Assessment Tool, *n*—femoral neck, it—intertrochanteric region, tot—total proximal femur.

The osteodensitometry analysis revealed a declining trend in BMD of the proximal femora and femoral neck in the ALC and CAA groups compared to control individuals (*p* > 0.05, Figure 2). Moreover, a declining trend in examined patient groups was noted for T and Z scores. The major difference was noted in lower BMD values of the intertrochanteric femoral region of individuals with ALC and CAA groups compared to healthy controls (*p* < 0.05, Figure 2). In addition, the osteodensitometry analysis of lumbar vertebrae showed lower T score and a tendency to declining BMD values in patients with the ALC compared to the CAA group, while the TBS analysis indicated that the vertebral micro-architecture was significantly better in controls when compared to individuals with ALC and CAA (Figure 2). A significantly higher risk of hip fractures and other major osteoporosis-related fractures was indicated by increased FRAX scores in patients with ALC and CAA compared to healthy controls (*p* < 0.05, Figure 2).

**Figure 3:**
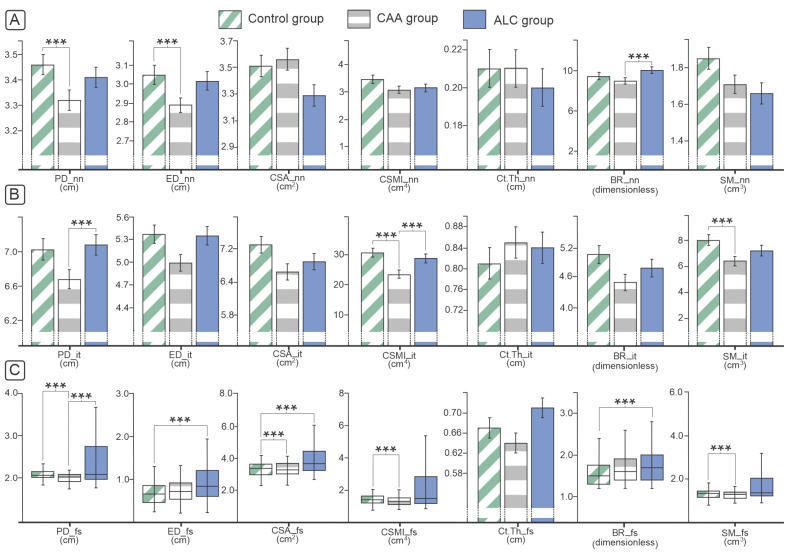
Femoral neck (**A**), intertrochanteric (**B**) and femoral shaft (**C**) geometry parameters in individuals with ALC and CAA. Analysis of covariance (ANCOVA) with Bonferroni posthoc correction was used for statistical analysis of the difference in BMI-adjusted parametric data (bar charts present data as mean ± standard error, *** marking significant difference). Kruskal-Wallis and Mann-Whitney tests were used to analyze intergroup differences in nonparametric data that did not display a normal distribution (data presented using box plot, *** is marking significant difference). Abbreviations: ALC—alcoholic liver cirrhosis, CAA—chronic alcohol abuse, PD—periosteal diameter, ED—endocortical diameter, CSA—cross-sectional area, CSMI—cross-sectional moment of inertia, Ct. Th—cortical thickness, BR—buckling ratio, SM—sectional modulus, nn—narrow neck femoral region, it—intertrochanteric femoral region, fs—femoral shaft.

Our analysis of geometrical femoral neck and intertrochanteric parameters, conducted using HSA, revealed the most prominent differences in the CAA group (Figure 3). On the femoral shaft of CAA individuals, the most significant decline was noted in almost all investigated parameters (Figure 3), while the ALC group had significantly different PD, ED, BR, and CSMI values compared to the control group (Figure 3).

### 3.3. Bone Turnover Biomarkers

In our sample, significantly higher serum β-CTX and OPG concentrations were verified in patients with ALC compared to the CAA and control individuals. RANKL concentrations were not significantly different between the investigated groups, while the lower RAKL/OPG ratio and IGF-1 concentrations were observed in patients with ALC and CAA compared to healthy controls (Table 2).

## 4. Discussion

Epidemiological data indicated that the frequency of bone fractures is between 5% and 20% of individuals with ALC [20,21], and one-third of these individuals have asymptomatic vertebral fractures [22]. Even though femoral fractures, compared with vertebral fractures, have a lower frequency in patients with liver cirrhosis and occur on average ten years after a vertebral fracture, it is important to note that femoral fractures in ALC patients are still more frequent than in the general population [8,23]. One of the useful tools in assessing which patients have a higher risk of developing nontraumatic bone fracture in the next ten years is the FRAX score, which is an additional factor in the clinical decision of which patients should be subject to osteological screening and treatment. In line with our findings, previous studies that have conducted FRAX analysis on patients with liver cirrhosis indicated that they have a higher fracture risk [24]. Also, a study conducted on Australian men over the age of 60 who consume three or more alcoholic drinks daily (30 g of alcohol or more) shows that they have higher FRAX scores for hip fractures and other major osteoporosis-related fractures [25]. The risk is significantly reduced if alcohol consumption is reduced to less than three drinks per day [25]. Although epidemiological data suggested a trend toward bone alterations, in our study, hip osteodenistometry only revealed significantly reduced intertrochanteric BMD in ALC and the CAA groups, compared to healthy age-matched controls (Figure 2). Also, TBS was considerably lower in our ALC and CAA patients than in healthy individuals. However, these observations do not indicate reliable diagnostic modalities for predicting bone fragility nor fully explain epidemiologic data about increased bone fragility in patients with ALC and CAA. While some studies described decreased hip BMD [14,16,18] and decreased lumbar spine BMD in ALC individuals [11,14,16], other studies reported the opposite findings, highlighting the absence of the difference between BMD values at the hip [11] or at the lumbar spine [26] in patients with ALC compared to healthy ones. Although in some studies, declined [17,27] or increased osteodenitometry parameters [28] were noted in individuals prone to chronic alcohol consumption, similar results of absence in BMD alterations in CAA individuals without liver cirrhosis, as noted in our study, were recently reported [29]. The possible reason for these heterogenic results in BMD changes in individuals with a history of heavy drinking and ALC could be related to certain interstudy differences (limited number of study participants, absence of consensus about the level of alcohol consumption for definition of heavy alcohol use, different approaches in defining the control group, absent data about smoking status and associated alcoholic related medical problems that are not taken into account [for example, pancreatitis, malnutrition, aromatase enzyme and estrogen deficiency, and vitamin D deficiency]) [14,15,16,17,18].

As a “gold standard” for assessing fracture risk, BMD measurements by DXA is a combination of cortical and trabecular bone mass analyses, but it is considered more reliable for measuring trabecular bone mass. To improve the estimation of cortical bone, we used HSA to assess femoral geometry in investigated groups. The majority of structural parameters were affected in patient groups, suggesting that cortical mechanical properties had significantly worse outcomes in our study. Therefore, neck BR, as one of the indexes of cortical stability, was significantly higher in patients with ALC and exceeded the “critical” value for increased bone fragility. In the intertrochanteric region, we found significantly lower CSMI and SM in ALC and CAA patients compared with the healthy control group. Based on previous studies, fracture resistance of the proximal femora is essentially determined by the CSMI, which is dominated by the contribution of the cortex [30]. Since the SM is an indicator of the bending resistance of the bone, reduced intertrochanteric SM indicated a lower resistance to fracture in CAA patients. At the femoral shaft, we observed a significant reduction in nearly all structural parameters (CSA, CSMI, SM, and BR) between patients with ALC and CAA and control individuals. Osteoporotic fractures are caused by both cortical thinning and trabecular bone loss. Still, in the literature, there is no consensus about the contribution of these two parts to bone fragility. Removing cancellous bone from the neck of the femur decreases the strength by less than 10% [30]. Our previous study noted severe ALC-induced microstructural changes, predominantly reflected in increased intertrochanteric cortical porosity, decreased intertrochanteric cortical thickness, and altered trabecular micro-architecture [31]. Significantly worse outcomes in structural parameters in ALC and CAA patients suggest bone strength degradation and increased susceptibility to femoral fracture in these individuals. Additionally, one of the studies performed on inducing chronic alcohol consumption in rats showed that even without altering BMD, alcohol consumption caused changes in the morphology and percentage of collagen in femoral neck trabeculae, which could make the bone more fragile [32].

Ethiopathogenetic mechanisms of bone loss in individuals with ALC and CAA are multifactorial and still insufficiently understood. Our data confirmed affected synthetic and excretory liver function in the ALC and CAA groups. Also, reduced testosterone synthesis was revealed in our ALC and CAA groups, which activated a negative feedback loop and subsequently caused LH to be secreted and SHBG to be synthesized in the liver. Increased SHBG concentration, along with albumins, binds testosterone, which affects an additional reduction of its free fraction [33,34]. Also, patients in both examined groups had lower vitamin D levels compared to healthy controls. These parameters could additionally contribute to bone loss in investigated groups. However, the expected negative feedback reaction of PTH was absent in our individuals and reduced PTH concentrations were noted in patients with ALC and CAA compared to the control group, which is in accordance with previous findings [14,35]. The potential reason for this finding could be that alcohol can lead to a transient and reversible decrease in PTH secretion, while the exact mechanism of hypoparathyroidism in patients with liver cirrhosis is not clear [14,35]. Besides these general mechanisms, systematic production of pro-inflammatory mediators (IL-1, IL-6, IL-17, and TNF-α) could contribute to bone loss in patients with ALC and CAA [6]. IGF-1 is synthesized in the liver and greatly influences bone metabolism by suppressing osteoblast apoptosis and stimulating osteoblastogenesis through stabilization of the Wnt/β-catenin pathway [36]. At the same time, IGF-1 has a negative effect on bone resorption via the RANKL/OPG pathway [37]. By decreasing the liver synthetic function and by progressive loss of growth hormone receptors on hepatocytes, synthesis and concentration of IGF-1 could also be reduced, especially in advanced stages of liver cirrhosis [38,39], which consequently could lead to bone deterioration [37], as noted in our study (Table 2). β-CTX is one of the most reliable biomarkers of bone resorption, which, due to its rapid response, can also be used to monitor the success of bisphosphonate therapy [40]. Increased β-CTX concentrations were previously noted in patients with liver cirrhosis. On the other hand, it is reported that moderate alcohol consumption suppresses osteocalcin and β-CTX in the serum, which indicates bone resorption decrease [41,42]. Our results are in accordance with the literal data, where significantly higher values of β-CTX were obtained in patients with ALC in comparison with the CAA and control group; while comparing the values of osteocalcin, no statistically significant difference was found between the groups. By binding to RANKL, OPG inhibits osteoclastogenesis, indicating that lower OPG values are expected in ALC and CAA patients. However, previous studies examining patients with CAA [43] and ALC indicated elevated OPG values, as we observed in our study [44]. The high OPG concentrations could be interpreted as a compensatory reaction to inflammatory-mediated bone resorption in ALC and CAA patients [45]. It is known that factors affecting RANKL formation also regulate the synthesis of OPG [46]. Ethanol per se [47] and liver cirrhosis, as a pro-inflammatory condition, promote the production of IL-6 and TNF-α, increasing the synthesis of RANKL and, consequently, promoting bone resorption [45]. Furthermore, the elevated RANKL concentrations could be interpreted as a consequence of the increased activity of osteoblasts and not only as a marker of bone resorption [48]. Therefore, a low RANKL value indicates reduced bone turnover, leading to poor bone quality and fragility. However, previous literature is not consistent about the RANKL concentrations in patients with liver cirrhosis. While certain data showed an increased RANKL value [44], others showed that despite the increased OPG concentrations, there was no difference in RANKL concentrations between patients with liver cirrhosis and healthy controls [43], which is in accordance with our data (Table 2). These findings could be partially explained by our study design, which included the patients without previous bone fractures and the initial stage of bone loss in ALC and CAA patients.

Apart from its main strengths, our study had the following limitations. This was a cross-sectional study, meaning we could not follow time-dependent bone deterioration in our individuals. Our study is also limited by the number of patients due to strict inclusion criteria and the fact that our research was conducted during the COVID-19 pandemic. Although we tried to control for the most likely confounding comorbidities, our results may be biased by some asymptomatic diseases and/or unreported habits (for example, smoking).

Our data indicated significant bone alterations in patients with ALC and CAA even before nontraumatic bone fracture occurred, but it is also indicative that current skeleton-assessing clinical methods are not fully reliable. These data suggest that several different examination modalities are currently needed to prevent nontraumatic bone fractures and to ensure adequate clinical management in each patient with ALC and CAA. Future studies should focus on developing a single reliable integrative clinical algorithm that can be used to predict and prevent the occurrence of nontraumatic bone fractures in patients with ALC and CAA.

## Figures and Tables

**Table 1:** The intergroup comparisons of biochemical blood parameters.

	ALC Group (Mean ± SE)	CAA Group (Mean ± SE)	Control Group (Mean ± SE)	*p* Value Overall	*p* Value ALC vs. CAA	*p* Value ALC vs. Control	*p* Value CAA vs. Control
PT (s)	16.99 ± 0.61	11.89 ± 0.12	11.50 ± 0.09	*p* < 0.001	*p* < 0.001	*p* < 0.001	1.00
Fibrinogen (g/L)	2.79 ± 0.14	3.98 ± 0.11	3.38 ± 0.12	*p* < 0.001	*p* < 0.001	0.015	0.007
Albumin (g/L)	36.47 ± 0.86	43.96 ± 0.39	46.56 ± 0.51	*p* < 0.001	*p* < 0.001	*p* < 0.001	0.015
Total bilirubin (μmol/L)	47.84 ± 7.65	8.72 ± 0.78	14.11 ± 1.17	*p* < 0.001	*p* < 0.001	*p* < 0.001	1.000
Direct bilirubin (μmol/L)	25.44 ± 5.19	2.68 ± 0.41	4.06 ± 0.33	*p* < 0.001	*p* < 0.001	*p* < 0.001	1.000
AST (U/L)	47.74 ± 4.78	31.18 ± 3.00	24.23 ± 1.76	*p* < 0.001	0.002	0.000	0.629
ALT (U/L)	32.61 ± 2.74	35.33 ± 3.92	32.37 ± 3.13	*p* > 0.05	/	/	/
ALP (U/L)	112.63 ± 7.48	70.89± 2.09	69.23 ± 2.47	*p* < 0.001	*p* < 0.001	*p* < 0.001	1.000
GGT (U/L)	95.04 ± 14.69	71.20 ± 8.28	30.44 ± 3.52	*p* = 0.001	0.279	0.001	0.036
Ca (mmol/L)	2.34 ± 0.02	2.40 ± 0.01	2.43 ± 0.02	*p* = 0.001	0.010	0.002	0.882
Ca^2+^ (mmol/L)	1.27 ± 0.01	1.30 ± 0.01	1.29 ± 0.01	*p* = 0.025	0.024	0.195	1.000
P (mmol/L)	1.07 ± 0.04	1.02 ± 0.03	0.89 ± 0.03	*p* = 0.004	0.720	0.003	0.034
Vitamin D (nmol/L)	31.48 ± 3.24	29.99 ± 2.58	45.93 ± 37.59	*p* = 0.002	1.000	0.009	0.003
PTH (pg/mL)	37.41 ± 3.55	36.82 ± 2.89	71.12 ± 5.26	*p* < 0.001	1.000	*p* < 0.001	*p* < 0.001
Osteocalcin (μg/L)	17.54 ± 1.71	17.27 ± 1.24	19.91 ± 1.25	*p* > 0.05	/	/	/
Testosteron total (nmol/L)	16.19 ± 1.27	19.13 ± 1.30	20.04 ± 1.48	*p* > 0.05	/	/	/
Testosteron free (nmol/L)	4.68 ± 0.84	7.83 ± 1.37	9.98 ± 0.96	*p* = 0.002	0.156	0.001	0.469
Estradiol (pmol/L)	164.05 ± 16.02	140.03 ± 9.09	131.22 ± 5.54	*p* > 0.05	/	/	/
LH (mIU/mL)	6.87 ± 5.13	6.67 ± 4.71	3.09 ± 2.59	*p* = 0.001	1.000	0.002	0.007
FSH (mIU/mL)	9.07 ± 1.40	8.92 ± 2.11	5.49 ± 0.64	*p* > 0.05	/	/	/
DHEAS (μmol/L)	4.46 ± 0.99	7.09 ± 1.03	4.28 ± 0.76	*p* > 0.05	/	/	/
SHBG (nmol/L)	69.82 ± 3.40	59.34 ± 3.77	44.54 ± 3.24	*p* < 0.001	0.111	*p* < 0.001	0.015

Analysis of variance with the Bonferroni posthoc test was used for statistical analysis of the intergroup difference. Abbreviations: SE—standard error, ALC—alcoholic liver cirrhosis, CAA—chronic alcohol abuse, PT—prothrombin time, AST—aspartate aminotransferase, ALT—alanine aminotransferase, ALP—alkaline phosphatase, GGT—gamma-glutamyl transferase, PTH—parathyroid hormone, LH—luteinizing hormone, FSH—follicle-stimulating hormone, DHEAS—dehydroepiandrosterone sulfate, SHBG—sex hormone binding globulin.

**Table 2:** Bone turnover biomarkers in ALC and CAA individuals.

	ALC Group	CAA Group	Control Group	*p* Value Overall	*p* Value ALC vs. CAA	*p* Value ALC vs. Control	*p* Value CAA vs. Control
β-CTX (pg/mL)	6202.10 ± 437.96	2819.23 ± 390.46	4224.12 ± 497.75	*p* < 0.001	*p* < 0.001	0.011	0.087
OPG (pg/mL)	391.88 ± 19.48	309.58 ± 20.54	252.59 ± 21.73	*p* < 0.001	0.014	*p* < 0.001	0.182
RANKL (pg/mL)	2584.00 [1171–5657]	2737 [1408–4980]	2701.79 [1408–7900]	*p* > 0.05	/	/	/
RANKL/OPG ratio	7.65 [1.43–21.02]	7.80 [3.27–23.50]	11.28 [5.11–27.49]	*p* = 0.003	0.502	0.003	0.004
IGF-1 (ng/mL)	38.60 [23.97–122.90]	35.31 [24.21–136.30]	52.72 [31.30–233.50]	*p* < 0.001	0.660	0.001	*p* < 0.001

Analysis of variance with the Bonferroni posthoc test was used for statistical analysis of the intergroup difference in data with normal distribution. Parametric data are presented as mean ± standard error. Kruskal-Wallis and Mann-Whitney tests were used to analyze intergroup differences in nonparametric data that did not display a normal distribution (data presented as mean [min-max]). Abbreviations: ALC—alcoholic liver cirrhosis, CAA—chronic alcohol abuse, β-CTX—beta-C-terminal telopeptide, RANKL—receptor activator of nuclear factor kappa beta (NFkB) ligand, OPG—osteoprotegerin, IGF-1—insulin-like growth factor 1.

## Data Availability

Data that support findings generated during this study are part of the first author’s PhD thesis and are available from the corresponding author, (D.D.), upon justified request.
